# Feasibility study of prospective gated tomosynthesis using a wide-angle carbon nanotube enabled, stationary digital chest tomosynthesis scanner: In-vivo evaluation in a porcine subject

**DOI:** 10.1371/journal.pone.0350286

**Published:** 2026-06-05

**Authors:** Alex Billingsley, Christina Inscoe, Jianping Lu, Otto Zhou, Yueh Z. Lee

**Affiliations:** 1 Joint Department of Biomedical Engineering, The University of North Carolina at Chapel Hill and North Carolina State University, Chapel Hill, North Carolina, United States of America; 2 Department of Physics and Astronomy, The University of North Carolina at Chapel Hill, Chapel Hill, North Carolina, United States of America; 3 Department of Radiology, The University of North Carolina at Chapel Hill, Chapel Hill, North Carolina, United States of America; PLOS ONE, UNITED KINGDOM OF GREAT BRITAIN AND NORTHERN IRELAND

## Abstract

**Background:**

Tomosynthesis offers a valuable alternative to computed tomography (CT) in longitudinal studies for dose reduction and applications where CT is not an option. This study aimed to demonstrate the performance of physiological gated tomosynthesis on the wide-angle-, carbon nanotube (CNT) x-ray based, stationary digital chest tomosynthesis system (s-DCT). Physiologic gating allows for image quality improvements by reducing motion blur and expands the potential applications.

**Methods:**

Prospective physiological gating was implemented on the system and a porcine subject was imaged under various gating strategies. Respiratory-only, cardiac-only (breath-hold), and dual gated scans were acquired along with breath-hold-only and free-breathing scans to study independent and joint gating performance. Quantitative analysis was performed by measuring full-width-at-half-maximum (FWHM) from the derivative of line profiles across the edge of the heart wall and diaphragm.

**Results:**

Dual gating had the highest image quality performance both qualitatively and quantitatively. No significant difference was observed between expiratory phase respiratory gating and breath hold, and during breath hold, cardiac gating significantly improved heart wall FWHM. All gated acquisitions performed better overall than free breathing.

**Conclusions:**

This study successfully demonstrated physiologic gating on the second-generation s-DCT system with high-quality images and imaging times similar to predicted.

## Background

Heart disease, the leading cause of death in the United States, claims 659,000 lives annually, accounting for 1 in 4 deaths [1,2]. Lung cancer causes the most cancer-related deaths in the United States [3]. Mortality rates are higher in rural areas due to limited access to high-quality specialized imaging outside of urban areas. Computed tomography (CT) is the standard of care in lung cancer and cardiovascular disease, but staffing, expense, and upkeep make access difficult in rural or underserved areas. Constant upkeep, acquisition of expensive dedicated equipment, and experienced technologists are less commonly available in underserved areas.

Many studies have investigated a pseudo 3D imaging modality, chest tomosynthesis, for evaluation of lung lesions [4,5]. The studies concluded that tomosynthesis, albeit short of CT performance, exhibits increased sensitivity compared to radiography and could be beneficial in routine lung nodule evaluation, replacing or complementing chest radiography, with potential in various thoracic imaging applications. Tomosynthesis scans are typically completed in 10s under breath hold [6,7]. However, physiologic motion, even during breath hold, can contaminate the images [8,9].

Incorporating physiologic gating to reduce motion blur could substantially improve image quality and expand the clinical use cases [10]. While other research groups have simulated gated tomosynthesis, prospectively gated chest tomosynthesis has not been demonstrated elsewhere [11–14].

Traditional tomosynthesis is constrained by the physical motion of the x-ray source, which can be eliminated entirely using stationary multisource x-ray tubes [15,16]. Carbon nanotube (CNT) x-ray technology enables multi-source x-ray tubes with closely packed focal spots. This enabled stationary tomosynthesis, demonstrated previously [17 ^–^ 18]. Simplicity of x-ray firing on demand, without the need for motion during tomosynthesis scanning, makes triggering x-rays based on physical or physiologic events during acquisitions straightforward [10,19,20]. The goal of this study is to demonstrate prospective respiratory and cardiac gated imaging using tomosynthesis.

## Methods

The wide-angle s-DCT system comprises a linear CNT x-ray source array, flat panel detector, electronic control equipment, and radiolucent patient bed. Microcontroller software was custom-developed for x-ray source control and physiologic gating. All experimental protocols were approved by the ethics committee of The University of North Carolina at Chapel Hill under Protocol #23–061.0. A single porcine subject (n = 1) was used in this study. The subject weighed 40 kg and had a thoracic diameter of 20 cm, approximately 63% the size of an average adult human male [21–23]. Porcine experiments were performed under an approved Institutional Animal Care and Use Committee (IACUC) protocol and all methods complied with the ARRIVE and American Veterinary Medical Association (AVMA) guidelines. All methods were performed in accordance with the relevant guidelines and regulations. Veterinary supervision of the study and procedures were performed by the institutional Division of Comparative Medicine. The use of animals during this study was in accordance with the approved protocol and standard operating procedures established by the Animal Care and Use Committee of the University of North Carolina at Chapel Hill, which strictly adheres to the Animal Welfare Act to assure limited discomfort and pain. All procedures were performed with the assistance of the Division of Comparative Medicine. Anesthesia and sedation were administered by Division of Comparative Medicine veterinary services to minimize discomfort and distress. General anesthesia was obtained with TKX Solution (Telazol 100 mg/ml: Ketamine 50 mg/ml; Xylazine 50 mg/ml) at 1 ml/50 lbs IM, and the anesthesia plane was maintained with Isoflurane (2–5%). The subject was continuously monitored during the procedures. The subject was euthanized with an intravenous overdose of pentobarbital (>100 mg/kg) administered by DCM veterinary staff, followed by thoracotomy.

### Device description and imaging protocol

**Fig 1:**
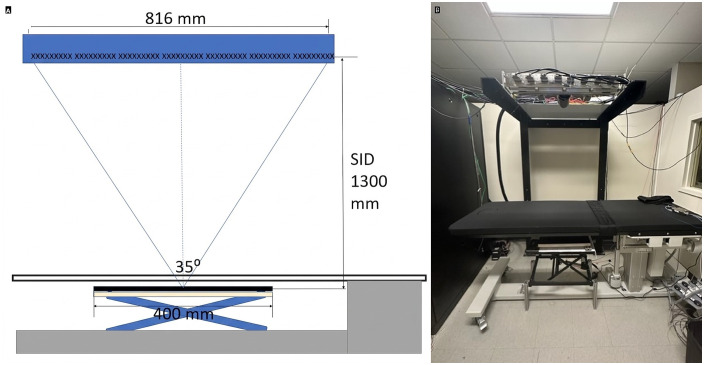
s-DCT imaging system design and matched picture of the actual system. A radiolucent patent bed is positioned below the x-ray source with the 40 cm detector placed on a scissor lift directly underneath the patent bed.

The s-DCT scanner design includes a source to image (detector) distance (SID) of 1.3m, 35-degree angular span, and 63 x-ray sources distributed over 816 mm (NuRay Technology Co., Ltd., Changzhou, China). Fig 1a shows a schematic demonstrating design values. A Varian 4030X detector (Varian Medical Systems, Palo Alto, California), served as the image receptor in the s-DCT imaging system. The detector has a 0.194x0.194mm pixel size and was operated at 10 frames per second (fps) at full resolution. Tube power, sampling, and angular span on the second-generation system was improved from prior s-DCT system originally with 5mA, 80kVp, 29 projections, and 12-degree angular span [18,24–28]. The wide-angle x-ray tube, developed by NuRay, has 63 sources divided into 7 groups of 9 sources, with inter-group spacing of 24 mm, and otherwise, an inter-source spacing of 12 mm. Fig 1b demonstrates the actual s-DCT system. The focal spot size is IEC 0.8, and each focal spot was operated using a 2.0ms pulse width at a cathode current of 24.97 + /-0.07mA, resulting in a tube current of 20.4 + /-0.6mA. Average mAs per projection was 0.039mAs (2.36mAs total). Cathode output consistency is dynamically enforced by the electronic control system, and anode current is measured with every scan to monitor individual source performance over time. X-ray tube window filtration is 0.2 mm of stainless steel, with a measured first half value layer of 5.17 ± 0.08 mm Al at 120kVp and total filtration equivalent of 4.39 ± 0.1 mm Al.

Scans were performed at 120kV with 60 projections over a 35-degree angular span. Total entrance dose was 0.2mGy.

### Physiological gating

**Fig 2:**
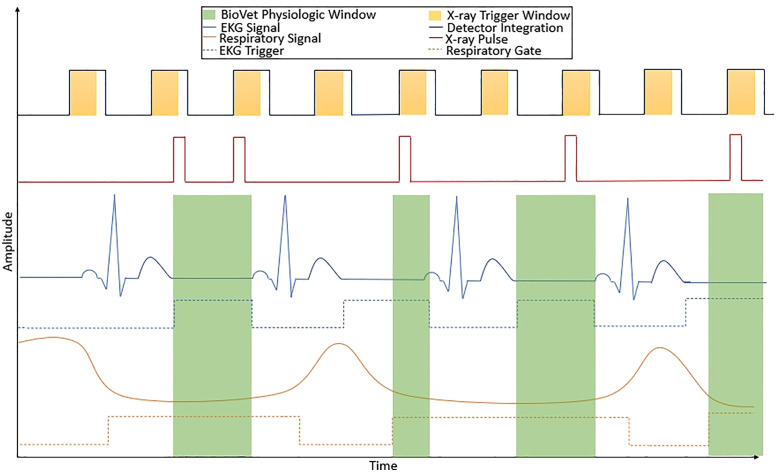
Physiologic gating timing diagram. Demonstrates ability of firing multiple x-ray pulses within a single gating event by utilizing the signal output (BioVet Physiologic Window) set with delays and time windows in the BioVet software after the point of exceeding a set threshold. Within the first physiological window, two x-ray pulses were triggered.

A BioVet (m2m imaging, Cleveland OH) was used to generate physiologic event triggers from respiration and electrocardiogram (EKG) signals. EKG was collected using leads, and respiration was measured using a pneumatic bellows (pillow-type) sensor positioned at the level of the diaphragm. The BioVet output can deliver triggers derived from the EKG or respiratory signals, or the logical ‘AND’ of both signals. This is shown by the green rectangles in Fig 2, which demonstrates the timing diagram for simultaneous cardiac and respiratory gating. For x-ray firing during gating, a single source per detector frame can be fired at any time the x-ray trigger window is active and the physiologic TTL signal is logical true. In turn, this enables multiple x-ray pulses to be triggered if the physiological window is sufficiently long (Fig 2, first physiological window).

X-ray control was handled using software developed on the Arduino Uno microcontroller. The user interface for controlling the scan and interacting between PC and microcontroller was developed using LabVIEW (National Instruments, Austin, TX).

Microcontroller operation in gated acquisition detects rising edge triggers from the integration inputs, then keeps track of the time elapsed following the rising edge trigger. If the time elapsed is less than the difference between integration time and x-ray pulse width, and the physiologic pin state is high, then it outputs an x-ray pulse. Otherwise, the Arduino continues to loop, until all cycles and x-ray pulses are acquired.

### Imaging study

Scans were acquired under free breathing (no physiologic gating), under mechanical ventilation breath holds, all with and without cardiac gating (Table 1). Respiratory gated scans were performed and compared to breath hold, using breath hold as standard for comparison to evaluate performance of the respiratory gating protocol. Respiration gating was performed at two different phases of the respiratory cycle, maximum inspiration and during the expiratory plateau using two different respiratory gating windows, 200 msec and 1500 msec, respectively. In general, maximum inspiration is more useful for lung parenchyma imaging while cardiac imaging can utilize the wider window of the exhalation plateau. Short respiration acquisition windows may also decrease the motion between the acquired projections, but longer windows increase the number of projections that can be obtained while also reducing scan time.

**Table 1:** Scans performed and associated premise.

Description	Premise
Ungated	Baseline comparator of all studies, free breathing without physiologic gating
breath hold	Standard for evaluation of respiratory gating alone
inspiration – short respiratory window	Protocol evaluation during inspiration for respiratory gated performance, motion-free peak inspiration is short, requiring shorter physiologic gating window
expiration – long respiratory window	Protocol evaluation during inspiration for respiratory gated performance
breath hold, contrast enhanced	Baseline for cardiac gating only – motion contaminated with (ideally) no impact from the lungs
breath hold + cardiac gating, contrast enhanced	Evaluation of cardiac gating alone with vessel emphasis as is relevant for when cardiac gating would be valuable
cardiac + respiratory gated	Dual gated evaluation for combination effects for respiratory and cardiac gating

Two contrast enhanced scans were also performed under breath hold to evaluate the cardiac gated protocol by itself. Use of contrast improved vessel visualization for qualitative performance. 35 mL of Omnipaque (350 mgI/mL) was manually administered intravenously through an ear vein at approximately 4 ml/second followed by a 20 mL saline flush. The scan was initiated immediately after contrast administration.

The cardiac and respiratory dual gated images were evaluated to observe combined effects on image quality. The gating windows used in the dual-gated cardiac and respiratory gated scan were 1500ms and 125ms for the EKG and respiratory signals, respectively. An independent evaluation of respiratory and cardiac gated scans was undertaken to determine the standalone effectiveness of each gating protocol, distinct from evaluating only the dual gated protocol. This approach aimed to reveal the specific contributions of respiratory or cardiac gating in isolation, addressing the uncertainty inherent in assessing solely the dual gated protocol.

### Theoretical framework for scan time consideration

To accurately evaluate the feasibility and performance of the CNT-enabled stationary digital chest tomosynthesis (s-DCT) system, it is essential to understand the theoretical considerations for scan times under different acquisition protocols. Estimated scan times are influenced by several factors, including detector frame rate, heart rate, x-ray pulse width, and respiration rate.

For conventional systems with moving x-ray tubes, the scan time is limited by mechanical constraints, such as the translation speed and stopping time of the X-ray source. In contrast, the s-DCT system, with its stationary array and electronic control, eliminates these mechanical limitations, allowing for more flexible and efficient prospective gating.

Gated scan time on the s-DCT scanner can be estimated. For example, for a normal human scan, assuming 90 BPM (upper limits of normal), 25% of cardiac cycle is diastole, 10 FPS (detector limit), 60% of the cardiac events are aligned with a respiratory event due with 60% of respiratory cycle as expiration, 58ms integration time, and 2ms pulse width, the total scan time for 60 frames can be estimated as follows:

Average number of frames acquired per cardiac cycle during cardiac gating:


$$\begin{array}{cc} & \left(\left(\frac{\text{1}}{\text{90}bpm}\times \frac{\text{60}s}{\text{1}\;min}\times \text{25}\%\;diastole\right)+\text{0}.\text{058}s\;integration-\text{2}\times \text{0}.\text{002}s\;xray\;pulse\;width\right)\\ & \times \text{10 }frames/s=\;\text{2}.\text{2}\text{ frames per cardiac cycle} \end{array}$$ (1)

Then incorporate the respiratory cycle:


$$\begin{array}{cc} & \frac{\text{2}.\text{2}\;frames}{cardiac\;cycle\;("heart\;beat")}\times \frac{\text{9}\text{0}\;heart\;beats}{\text{1}\;min}\times \frac{\text{1}\;min}{\text{6}\text{0}s}\times \text{6}\text{0}\%\;respiratory-cardiac\;alignment\;=\\ & \;\text{1}.\text{98 frames per second }(\text{cardiac and respiratory gating}) \end{array}$$ (2)

And compute the total imaging time:


$$\begin{array}{c} \text{6}\text{0}\;frames\;\times \text{1}\;s\;/\;\text{1}.\text{9}\text{8}\;frames\;=\text{ 30}.\text{3}\text{ seconds} \end{array}$$ (3)

This theoretical formulation was validated through simulation, implemented in Python. An algorithm was devised to compute the average frames per cardiac event after considering relative timing offsets between the x-ray firing window of detector integration and cardiac physiology. The detector and cardiac cycle are asynchronous; the number of valid frames per diastolic event depends on the relative timing offset. The algorithm generates firing window start and end times for several consecutive detector integration periods. The viable diastolic window, defined as the diastolic duration minus one pulse width, is then shifted in increments from zero to one full detector frame period. Enough consecutive detector frames are evaluated to ensure the diastolic window cannot extend beyond the last frame at any offset. At each offset, the algorithm counts the number of frames where the firing window overlaps with the viable diastolic window. The offset resolution refines by an order of magnitude with each iteration, increasing from 100 to 100,000 sampled offsets, and remains true when parameters are varied. The simulation is used to determine the range of theoretical scan times given constant physiologic and scan parameters during imaging, which are compared against experimental results.

The stationary s-DCT system achieves higher efficiency with its multi-source X-ray tube and electronic control. This system can acquire frames without the delays associated with mechanical motion, making it capable of clinically appropriate scan times while maintaining near-identical radiation dose levels to ungated acquisition. The greatest limitation to scan time under current conditions being the detector frame rate; 100FPS would allow for breath holds and scans under 2.4s. Additionally, the theoretical heart rate limit exceeds physiological capabilities. Using a diastole period limited to the beam on time, the theoretical heart rate limit is calculated as follows:


$$\begin{array}{c} {\left((\text{2}ms\;pulse\;width)\times \frac{\text{1}s}{\text{1}\text{0}\text{0}\text{0}ms}\times \frac{\text{1}}{\text{2}\text{5}\%\;diastole}\right)}^{-\text{1}}\times \frac{\text{6}\text{0}s}{\text{1}min}\;=\text{ 7500 bpm} \end{array}$$ (4)

This is well above normal heart rates, even under exercise or pathophysiological conditions.

While the s-DCT system offers significant advantages in speed and clinical utilization under varied pathophysiological conditions, it is important to evaluate the potential and limitation of conventional mechanically translated tomosynthesis systems for comparison. The reliance on mechanical translation of the x-ray source introduces additional challenges in terms of gating and scan time optimization. Scan time estimations were performed for mechanically translated, step-and-shoot, tomosynthesis to highlight these challenges. An algorithm was devised, to create multiple instances of the start and end time of diastole and the fraction of integration period that can encompass the entire x-ray pulse. The timing of the detector, cardiac events, and scan initiation is asynchronous, with the detector continuously running to ensure clear frame acquisition before any x-ray pulse is fired and stable operation of the detector. This operation, also used in the s-DCT system, requires the simulation of multiple offsets between the physiological triggering windows and the detector’s integration periods; outside of active translation, an x-ray is fired immediately when integration and physiology overlap. The source movement is defined by time in motion, occurring immediately after each x-ray source pulse width, which initiates coincidence with the start of the overlapped period between the integration window and physiologic window. The number of samples for offset were increased logarithmically to find the convergent average scan time and frames per cardiac event. Symmetric acceleration and deceleration with a linear ramp, immediate translation after each x-ray fire with no delays, similar sampling to the s-DCT, and a step-and-shoot protocol were assumed. Vibration and engineering constraints were ignored. Scan parameters were varied to evaluate minimum performance requirements to aid the comparison against the untranslated, multisource geometry. Parameters loosely informed by existing commercial systems were considered but were limited due to the traditional continuous translation in chest tomosynthesis. Continuous translation has separate limitations.

Continuous scanning tomosynthesis in prospective gated imaging is theoretically possible, but it is either limited to one exposure per cardiac event or introduces angular sampling inconsistencies, which can degrade image quality and whose effects worsen with increasing speed. Due to these limitations, continuous scanning performance was not calculated in detail beyond estimating the speed required to complete a scan within a single diastole period.

### Image reconstruction

Images were reconstructed into 3 mm slice thickness at full in-plane resolution (0.194x0.194mm). Reconstruction was performed using the adaptive fan volume – simultaneous algebraic reconstruction technique (AFVR-SART) algorithm [29].

### Quantitative analysis

**Fig 3:**
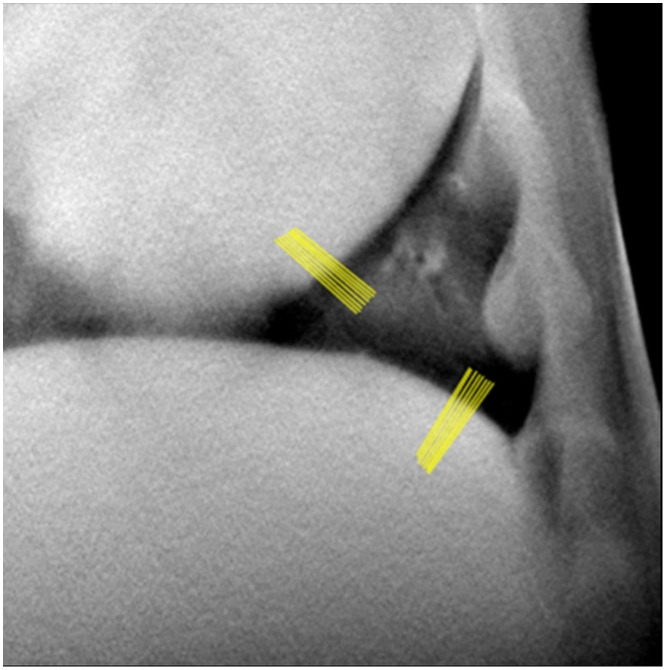
Line profile visualization as drawn for the heart wall and diaphragm for each tomosynthesis dataset. The example shown is from the breath hold, ungated, contrast enhanced dataset.

Line profiles were drawn across the heart wall and diaphragm edges, as an edge spread function for evaluation of gating performance based on edge sharpness. The edge spread function was differentiated to generate the point spread function for each profile, and the full width at half maximum (FWHM) was measured from each point spread function [30]. The same reconstruction algorithm and filtering parameters were used across all datasets, and no algorithm-specific tuning was performed. Therefore, differences in FWHM reflect acquisition conditions rather than reconstruction variability. Eight FWHM measurements were recorded for the diaphragm and cardiac wall to analyze gating performance as shown in Fig 3. A successful gated scan would have sharper edges, leading to a smaller FWHM. X-ray firing without proper gating, will lead to physiologic motion blur, which in turn blurs the edges of the anatomy in motion, and increase FWHM.

## Results

**Fig 4:**
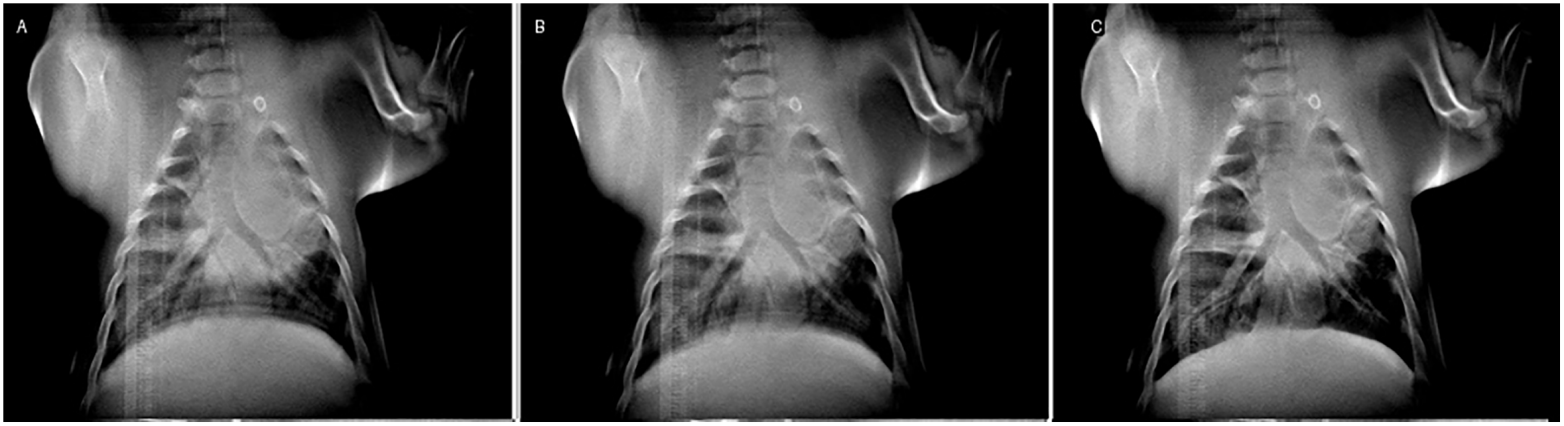
Respiratory gating compared to breath hold reconstruction images. A short physiologic window, capturing peak inspiration (**a**, 93.7s scan), a long physiologic window, capturing expiration (**b**, 10.4s scan), and a scan with a breath hold only (**c**, 6.3s). Slices of the lung emphasizing the bronchi were chosen for display. Clear improvement in motion blur across the diaphragm is observed in the two images at right, with the breath hold data showing the sharpest margins.

Table 2 lists each acquired dataset, the associated scan time and FWHM measurements. Fig 4 demonstrates the effectiveness of respiratory gating alone, showing the bronchi within the lungs for inspiration respiratory gating (a), expiration respiratory gating (b), and breath hold (c). Drastic differences are observed between the inspiration, expiration, and breath hold data, noted by the blurring of the diaphragm and bronchi. However, FWHM measurements in Table 2 show small differences between the edge sharpness of the diaphragm between expiration and breath hold data. Quantitative FWHM measures also show greater motion reduction from respiratory gating in the expiratory phase and the worst FWHM of the study. The average FWHM for the diaphragm changed very little, −1.3% and between breath hold and expiration gating, compared to a much worse +241% between breath hold and inspiration-based gating. The shorter inspiration phase gating window also led to substantially increased scan times, approximately 15x greater than breath hold and 10x greater than expiration triggering (Table 2).

**Table 2:** FWHM measurements of the diaphragm and heart under different imaging protocols. FWHM reported as value + /- s.d. (mm).

Description	Scan Time (s)	Heart Wall FWHM (mm)	Diaphragm FWHM (mm)
Ungated	6.3	4.52 + /- 0.99	4.51 + /- 0.29
breath hold	6.3	2.44 + /- 0.54	1.50 + /- 0.30
inspiration – short respiratory window	93.7	2.89 + /- 0.59	5.12 + /- 0.41
expiration – long respiratory window	10.4	2.67 + /- 0.60	1.48 + /- 0.35
breath hold, contrast enhanced	6.3	2.32 + /- 0.32	1.21 + /- 0.29
breath hold + cardiac gating, contrast enhanced	16.7	1.65 + /- 0.38	1.39 + /- 0.26
cardiac + respiratory gated	44.3	1.45 + /- 0.19	1.15 + /- 0.14

**Fig 5:**
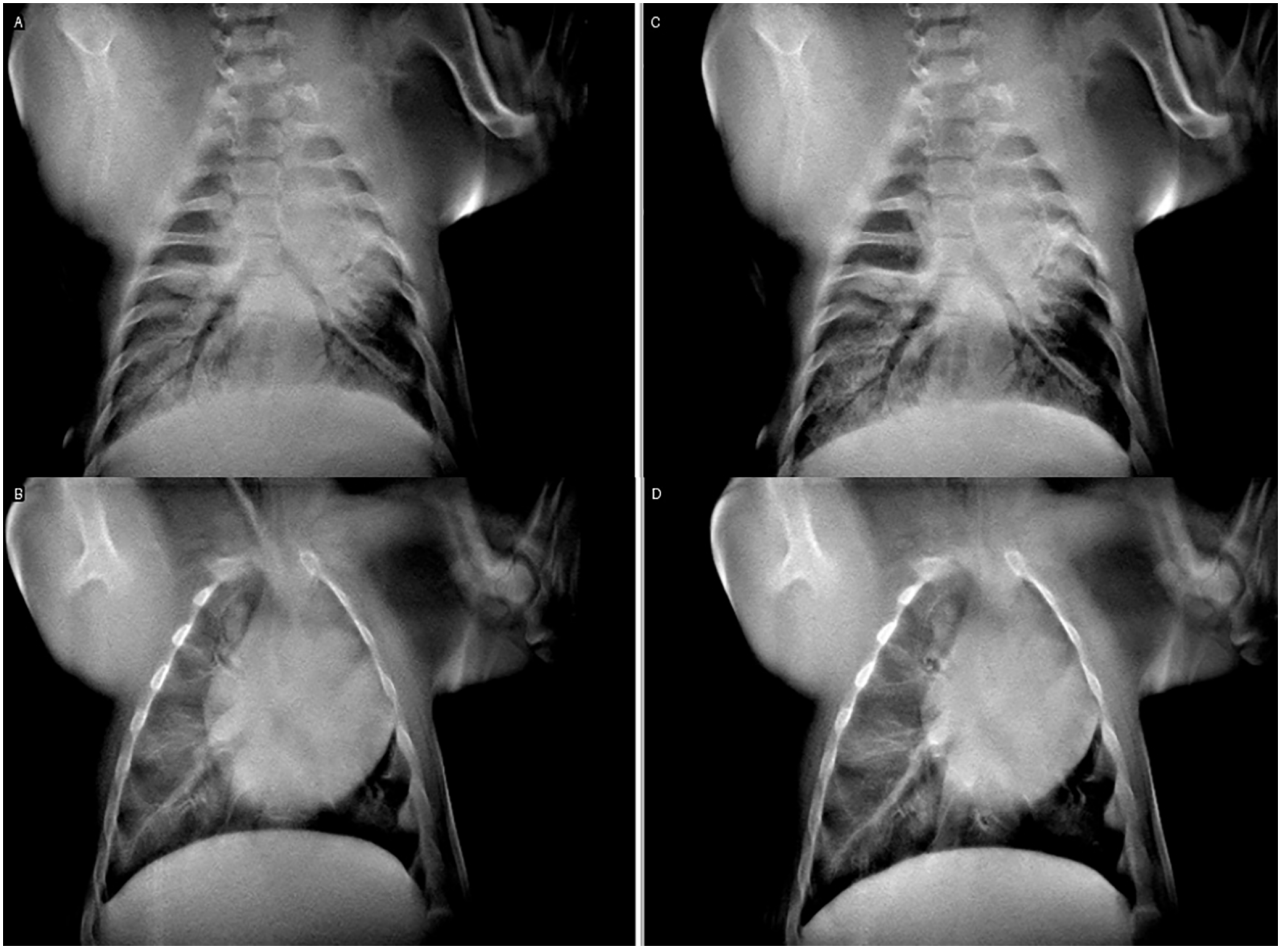
Post contrast scans utilizing a breath hold under mechanical ventilation without cardiac gating (a, b, 6.3s scan) and with cardiac gating (c, d, 16.7s scan). Clearer margins and distinguished vessels in the in-focus plane are observed in the cardiac gated images, despite the longer scan time.

Fig 5 shows two separate breath hold scans, one with cardiac gating (c, d), and one without cardiac gating (a, b). The top slices (a, c) in Fig 5 emphasize the lung bronchi, and the bottom slices (b, d) demonstrate the pulmonary vessels. Contrast was administered for these scans. The resulting images as demonstrated in Fig 5, showed improvement in visualization of vessels within and around the heart. These qualitative findings are complemented by quantitative results in Table 2, which demonstrate a substantially smaller average FWHM from the heart wall in the cardiac gated image set, 1.65 mm gated compared to 2.32 mm ungated. These results highlight the substantial improvement in motion artifact reduction with cardiac gating.

**Fig 6:**
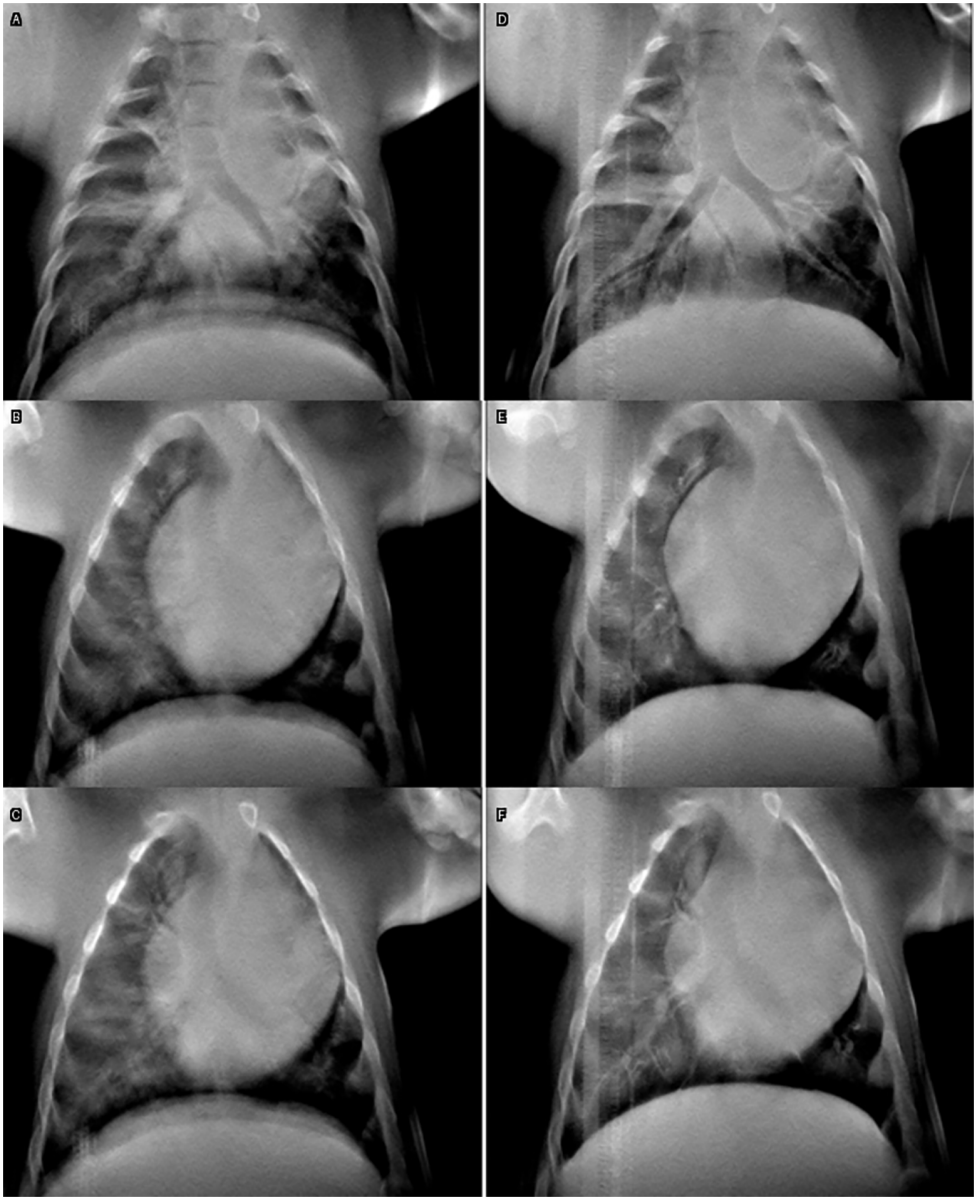
Images from different depth positions from the ungated, free breathing acquisition (a, b, c, 6.3s scan) and respiratory and cardiac gating (d, e, f, 44.3s scan) at the same depths. Blurring of the airways (a, d), heart (b, e) and diaphragm (c, f) is reduced in the gated images.

Fig 6 shows the ungated free breathing acquisition compared to the dual gated acquisition. The slices at the top of Fig 6 (a, d) were chosen to emphasize the image performance in visualization of the bronchi and trachea between free breathing (a, b, c) and dual-gated (d, e, f). Similarly, the other slices emphasize the heart (b, e) and surrounding vessels (c, f). Comparing the free breathing and dual cardiac and respiratory gated scans, we observed, as demonstrated in Fig 6 and Table 2, sharper edge preservation was obtained using the dual gated protocol across both the diaphragm and heart wall. The motion artifact from cardiac and respiratory motion both diminished substantially with the FWHM reducing from 4.52 mm (ungated) to 1.45 mm (dual-gated) for the heart, and from 4.51 mm to 1.15 mm for the diaphragm.

At a heart rate of 90bpm, the theoretical average scan time for cardiac and respiratory gating was calculated to be 30.3s (range: 22.2s – 33.3s). The actual scan time was completed in 44.3s. For cardiac gating only, scans were performed in 16.7s, close to the estimated 18.2s and falls within the range of expected values (range: 13.3–20s). These times were calculated using the algorithm that computes average frames per cardiac cycle. Although scan times were generally in line with expectations, slight deviations could be attributed to variations in heart rate and the precision of gating signals during the scan. Dynamic adjustments to the gating windows in response to physiological variations may further improve scan performance.

Comparing the s-DCT to the theoretical prospective gating performance of step-and-shoot tomosynthesis, substantial differences in performance are observed. Assuming all scan and physiologic parameters are the same from the 90bpm, 10FPS example in the methods, and a modest 0.3s per translation, a step-and-shoot acquisition results in a 66.7s acquisition time, due to the time required for the x-ray source to translate and settle between projections. The scan time would not improve further until the translation time becomes shorter than the diastolic period shortened by two x-ray pulse widths, which in this case is 0.1627s per translation. Simulations of step-and-shoot scanning methods showed that a 0.1-second translation time results in a 42.3-second scan at 10 FPS, or 33.3 seconds at 100 FPS, with the x-ray source accelerating at 0.567G. Further reduction to a 0.05-second translation reduced the scan time to 29.1 seconds at 10 FPS and as low as 10.7 seconds at 100 FPS, but required accelerations up to 2.2G. While higher acceleration stages are available, the increased scan speed comes with significant mechanical challenges and costs. In comparison, the gated stationary s-DCT system would achieve a scan time of 2.4 seconds at 100 FPS without the need for mechanical translation, providing a much faster and more efficient scanning option.

Continuous tomosynthesis scanning simulations at 90bpm and acquiring 60 x-ray projections, the system would need to complete the scan within the diastolic window of 0.166s to avoid highly inconsistent angular sampling or pedestrian (66.7s) scan times. Given the length between end sources of the s-DCT, the average velocity would be 4.92m/s with the framerate 362FPS. These frame rates are not available on current large-area detectors or commercial tomosynthesis systems [18,31]. The Shimadzu Sonialvision II is noted to operate at 15FPS [32]. While the speed is feasible, achieving such high accelerations and decelerations over short time periods would be a significant engineering challenge, especially when aiming to limit translation distance and scanner size, unless the variation in angular source density has minimal impact on image quality.

Cardiac gating with 250ms temporal resolution is required all cardiac CT. State of the art single source CT has 135ms temporal resolution, and dual source 66ms, beta blockers are still used in patients at heart rates above 65 bpm [33–37]. The heart rate limit for the stationary system was calculated to be 7500bpm, far exceeding physiological limits, even under stress conditions [38,39]. This indicates it is capable of extreme heart rates without beta blockers or performance degradation, using currently available components and technology, unlike the limited mechanically translated versions, demonstrating the potential of the s-DCT for use in cardiopulmonary imaging applications.

## Discussion

Prospective physiological gating on the wide-angle s-DCT scanner was successfully implemented. Our approach of systematically exploring the different gating strategies also enables us to separate the relative contribution of each approach to improving image quality. This is important in understanding how one might perform motion-free chest or cardiac imaging depending on the imaging needs or ability for a subject to follow instructions.

### Respiratory gating

Freezing respiratory motion in thoracic scans is a crucial component of improving image quality. For patients who are able to have more normal lung function and are able to follow directions, a simple “breath hold” command is usually sufficient to minimize respiratory motion with shorter scans. However, for patients who are in respiratory distress, unable to follow commands or intubated, performing breath-hold imaging is impossible. Thus, physiologic respiratory gating assists imaging of pediatric subjects, patients with chronic lung disease, and others who are unable to hold their breath [17]. Respiratory gating can also be performed at both maximum inspiration or during the exhalation plateau. During maximum inspiration, the time at peak volume and corresponding available imaging window is typically short, but the increased lung volume is important in evaluating lung pathology. The exhalation plateau usually is significantly longer, which increases the available time window for x-ray pulses. Expiration based gating showed improvements in edge preservation, although not completely effective at eliminating respiratory motion artifacts when qualitatively compared against the breath hold. Inspiration window gating paradoxically had the worst blur of the diaphragm, likely related to a poor max inspiratory gating trace signal and the extended scan time. The quantitative FWHM results demonstrated no significant difference between breath hold and expiratory phase gating, against much worse inspiratory gated images.

Of note, the expiration window is generally long and stable – roughly 60% of the breathing cycle. Expiration gating is simpler, reducing blur with faster scan times. Capturing inspiration requires a much shorter physiologic window, as peak inspiration holds a single physical state for a smaller relative time. A more precise piezoelectric or other respiratory transducer type may also improve the selection of the optimal inspiratory time window. We selected the exhalation phase imaging for subsequent use in our respiratory and cardiac gated imaging experiments. In general, if the focus of imaging is the heart, evaluating lung parenchyma is also less critical.

### Cardiac gating

Cardiac CT is performed using physiological gating in order to “freeze” the heart for improved structure visualization [40,41]. In prospective gated cardiac CT, a physiologic window is generated for x-ray output during the diastole period of the cardiac cycle. X-rays are fired during this time period to acquire all projections with the heart in the same phase. Imaging studies are typically performed under breath hold. A typical maximal heart rate of 60 BPM can be imaged with cardiac CTs before administration of beta-blockers are needed to pharmacologically slow the heart rate.

We first explored cardiac wall motion with and without cardiac gating under breath-held conditions, which would be equivalent to a clinical situation where a patient needs cardiac imaging but can follow breath-hold commands. The results show the cardiac gating scheme worked as desired, with reduction in cardiac motion artifact, demonstrated by visualization of vessels, and quantitatively better edge preservation.

### Dual cardiac and respiratory gating

For patients unable to follow commands or those with poor respiratory function, dual respiratory and cardiac gated imaging may be the only approach to obtain motion-free cardiac images. This also holds true for pediatric imaging. Breathing artifacts are often an issue in pediatric single-source CT, although comparably limited in dual source CT resultant from scan time differences for the completion of entire volumes during scans performed under breath hold [42]. Though seemingly straightforward in implementation, simultaneous gating is not often performed in human studies, likely due to the extended scan times. Dual gating has been common in CT for mouse studies using a micro-CT scanner [19,43].

The dual gated result is as anticipated, given the flexibility of the CNT x-ray system and indicates successful gating. Overall, the FWHM values were the lowest in our study, and despite the single subject study, strongly suggest the importance of both high quality respiratory and cardiac gating in obtaining motion minimized images.

Additional improvement in motion reduction was noted from gated studies. Respiratory gating reduced FWHM in heart wall measurements compared to free breathing, and dual gating further improved image quality as demonstrated by the narrowest FWHM across the measurements. These are not unexpected as the heart is attached to the lungs at its base, and motion in the heart or lung may result in motion in the other organ.

Of note, dual gating exhibited superior motion control over breath hold acquisitions. Breath holds are commonly used in clinical imaging, either through verbal commands to subjects or manipulation of the ventilator. The ventilator-controlled breath hold maintains a fixed air volume in the ventilator but through leakage of the air and other physiological mechanisms, the lung volume may slowly diminish during the paused breath. Lung volume was observed to decrease between breath held projections, especially apparent in longer scans. This is also known to happen in ventilated human subjects during extended breath holds; persons under anesthesia often experience atelectasis, from which leads to the observation of this phenomenon [44,45]. The improvement demonstrated by dual gating as compared to breath hold imaging is substantial adding an additional layer of image quality control in circumstances where a longer scan is required or perfect breath hold is unlikely or impossible.

### Scan time

Our results demonstrate the wide range of potential scan times with the different acquisition protocols. Estimated scan times will vary depending on detector frame rate, heart rate, x-ray pulse width, and respiration rate. Once the gating scheme implementation is optimized, the major factor limiting speed is detector frame rate. In the simulated and observed scan times, variation perceived in heart rate and breathing should be minimal. However, given the heart rate is variable, compensation may be necessary. Parameter compensation could be dynamically performed in-scan by scaling the physiologic cardiac and respiratory windows proportional to a moving average of the heart and respiratory period, respectively.

Faster x-ray detectors could significantly improve scan times on the s-DCT system, proportional to the improvement in frame rate (original scan time × 10fps / new fps) [31]. CNT multibeam x-ray sources can achieve much higher frequency acquisitions [46].

The ease of successful prospective physiologic gated tomosynthesis imaging is attributed to the simple control of cold cathode x-ray sources and CNT multi-beam x-ray technology. The multisource x-ray tube allows gated acquisitions without any dependence on step-and-shoot protocols, source motion control or translation speed limitations. High quality imaging can be performed at any perceivable human heart rate or respiratory rate, and there is no need for pharmacological reduction, as is often performed in cardiac gated CT [33,34]. To perform prospective gated imaging on a traditional tomosynthesis system, many of which acquire 60 projections or more, a step-and-shoot acquisition is theoretically possible, but at 90bpm, assuming a 0.3s translation per source is achieved, the average scan time of 66.7s using dual gating is possible. The issue with conventional systems is a single source in physical translation; there is a constant limitation to one frame per cardiac event, assuming an inability to translate, come to a stop, and fire an x-ray pulse within the duration of diastole at most heart rates. The 0.3s per translation time is slightly larger than stress diastolic percentages at a 60bpm heart rate. Unlike the stationary tomosynthesis system, in a conventional system in a prospective gated acquisition (step-and-shoot), scan time cannot be improved by using faster detectors, without substantially faster translations, while the physiologic limits on scan time and acquisition persist. Continuous motion would likely lead to inconsistent angular sampling and associated issues in the reconstruction result. This would be especially true if the heart rate varies at all during the acquisition. Alternatively, retrospective gating could be done in a continuous motion manner, but would remove much of the radiation dose benefits [11,12]. Alternative approaches have also explored mitigating motion artifacts in tomosynthesis by retrospectively excluding projections affected by breathing motion; however, this reduces the available projection data and has shown limited or inconsistent improvements in image quality, with potential increases in noise and reconstruction artifacts [47]. Continuous motion tomosynthesis introduces additional blur due to focal spot motion along the direction of tube travel, varying with source velocity and pulse duration [48]. This can further limit spatial resolution and impose constraints on exposure timing. Although flying focal spot techniques can compensate by maintaining an effectively stationary focal spot during acquisition, these approaches introduce additional system complexity and still rely on mechanical motion [49]. They do not directly address physiologic motion at acquisition time or enable dose-efficient prospective acquisition strategies. The stationary linear source array makes gated acquisitions possible in clinically appropriate scan times with near identical dose to ungated acquisition.

FWHM enables quantitative resolution measurement of the reconstructed anatomy in-motion [30]. Similar measures have been implemented to characterize gating based on edge strength, referenced as “Edge Response Width (ERW)” [50]. ERW measured raw edge width (or sharpness) direct from images, where FWHM uses a transformation of the line profile to measure the edge sharpness and blurring. The FWHM is another means well utilized for other purposes and quantifies resolution, which has proved pertinent in the development of the current standard-of-care MDCT based cardiac gated CT. The data as presented here shows both qualitatively, and quantitatively, the power of prospective respiratory, cardiac, and dual gating using stationary source arrays in the low-dose s-DCT scanner.

### Radiation dose

Radiation dose is of concern in children and longitudinal studies. Prospective gating has the ability to provide the image quality improvement of gated acquisition, without the increased dose from retrospective acquisition. Retrospective gating tomosynthesis is possible, but results in higher patient dose [51]. As we have demonstrated, the same prospective gating principles from other modalities can be applied to tomosynthesis, and is trivial with CNT x-ray sources [10,17,19,20]. Prospective gated tomosynthesis provides the benefit of “freezing” the heart and/or lungs, but also helps to minimize radiation exposure compared to retrospective or computed tomography in routine imaging. Gated scans use a measured entrance dose of 0.25mGy, which falls within reported values for chest radiography. Published studies commonly report entrance doses on the order of ~0.1–0.3mGy, although higher values (~0.3–0.9mGy) have been reported depending on imaging technique, system configuration, and acquisition parameters [17,52–54]. Other studies with systems of similar operating parameters have demonstrated effective dose of tomosynthesis in the range ~0.13−0.2mSv, compared to ~0.05–0.1mSv for chest radiography and 2-3mSv on chest CT [55–57]. A more recent report showed specifics for lung cancer screening, 1.5mSv for CT and 0.1mSv for chest radiography [58]. There is approximately an order of magnitude reduction in dose using the second generation s-DCT tomosynthesis system compared to CT. The reduction in radiation is of great importance as shown by the recent push to focus on the principle “As Low As Reasonably Achievable” (ALARA) in radiation exposure, and thus the tomosynthesis system would offer the potential to reduce radiation dose.

### Future studies

Heart disease is the number one killer in the US, and lung cancer, among cancers, causes the most deaths. Mortality is worsened in rural areas due to access to care, and tomosynthesis offers a cheaper, low dose, alternative to CT with the ability to service these poorly served areas, with improved performance compared to radiography. Successful respiratory and cardiac imaging allows for image quality improvements, larger breadth in addressable populations, and expands the number of use cases for tomosynthesis toward more advanced imaging techniques [20,59] Tomosynthesis has been limited in image quality and performance compared to CT, although offering a lower dose, cost effective alternative that is feasible in low resource settings [60]. Although the power of this study is low with a single porcine subject (n = 1), the qualitative and quantitative results demonstrate significant improvements and success of both respiratory and cardiac gated tomosynthesis imaging implementation and protocol. In addition to these quantitative and qualitative assessments, future studies could incorporate visual grading analysis to evaluate image quality in a clinically relevant, observer-based manner [61]. The experiments demonstrated we can eliminate the physiologic motion blur with proper gating parameters, in reasonable scan times. Gating quality is also highly dependent on our gating signal source quality, and acquiring higher quality respiratory traces will further serve to improve image quality.

Additional studies will be conducted to demonstrate the power and expanse of various applications within tomosynthesis using gated protocols. Through gated tomosynthesis, more opportunities for new or improved, advanced or clinically difficult, applications in chest tomosynthesis imaging become possible, such as calcium scoring, lung nodule evaluation near the heart, pediatric cystic fibrosis evaluation, and image-guided therapy and intervention [17,20,59,62–64].

## Conclusion

Physiologic gating was successfully demonstrated with the second-generation s-DCT system. Across all image sets, the gating performance has been demonstrated successful for both respiratory and cardiac application. The new gating protocol works effectively and produces high quality images in a fraction of the time of the first iteration s-DCT system. Porcine study results indicate success and potential for translation of gated studies in the clinic. When used clinically, we would be able to improve image quality in chest tomosynthesis applications. It would serve to be seen if gated tomosynthesis could offer an improvement on sensitivity to lung nodules, or other clinical measures of image quality.
